# Investigating Foreign Language Vocabulary Recognition in Children with ADHD and Autism with the Use of Eye Tracking Technology

**DOI:** 10.3390/brainsci15080876

**Published:** 2025-08-18

**Authors:** Georgia Andreou, Ariadni Argatzopoulou

**Affiliations:** Department of Special Education, University of Thessaly, 38221 Volos, Greece; andreou@sed.uth.gr

**Keywords:** ADHD, ASD, foreign language, eye tracking, word recognition, vocabulary

## Abstract

Background: Neurodivergent students, including those with Autism Spectrum Disorder (ASD) and Attention Deficit/Hyperactivity Disorder (ADHD), frequently encounter challenges in several areas of foreign language (FL) learning, including vocabulary acquisition. This exploratory study aimed to investigate real-time English as a Foreign Language (EFL) word recognition using eye tracking within the Visual World Paradigm (VWP). Specifically, it examined whether gaze patterns could serve as indicators of successful word recognition, how these patterns varied across three distractor types (semantic, phonological, unrelated), and whether age and vocabulary knowledge influenced visual attention during word processing. Methods: Eye-tracking data were collected from 17 children aged 6–10 years with ADHD or ASD while they completed EFL word recognition tasks. Analyses focused on gaze metrics across target and distractor images to identify percentile-based thresholds as potential data-driven markers of recognition. Group differences (ADHD vs. ASD) and the roles of age and vocabulary knowledge were also examined. Results: Children with ADHD exhibited increased fixations on phonological distractors, indicating higher susceptibility to interference, whereas children with ASD demonstrated more distributed attention, often attracted by semantic cues. Older participants and those with higher vocabulary scores showed more efficient gaze behavior, characterized by increased fixations on target images, greater attention to relevant stimuli, and reduced attention to distractors. Conclusions: Percentile-based thresholds in gaze metrics may provide useful markers of word recognition in neurodivergent learners. Findings underscore the importance of differentiated instructional strategies in EFL education for children with ADHD and ASD. The study further supports the integration of eye tracking with behavioral assessments to advance understanding of language processing in atypical developmental contexts.

## 1. Introduction

Neurodivergent learners, such as individuals with Autism Spectrum Disorder (ASD) or Attention Deficit/Hyperactivity Disorder (ADHD), often encounter cognitive, linguistic, and attentional challenges that can hinder the process of language development [1]. Research has shown that people with neurodevelopmental conditions experience language problems across various linguistic domains [2]. These linguistic difficulties frequently extend to the field of foreign language learning, with empirical evidence suggesting that individuals with language-related difficulties encounter persistent challenges in acquiring additional languages [3,4]. Despite this, research and educational interventions focusing on foreign language acquisition tend to prioritize typically developing (TD) children, with comparatively less attention given to neurodivergent populations [5]. Nevertheless, in research examining different educational programs and foreign language learning for students with ADHD and ASD, vocabulary acquisition is consistently identified as a key focus and often serves as the primary objective of the study. This focus is evident in both traditional teaching methods and technology-enhanced foreign language learning [6,7,8]. This happens because vocabulary has a vital role in learning a foreign language, as it affects not only reading and listening comprehension but also speaking and writing abilities [9,10].

Eye tracking technology has been widely used in research involving neurodivergent groups, mainly serving as a valuable tool for diagnosing and gaining insights into these conditions [11,12,13,14]. This technology has also become a prominent methodological approach for studying various language-related challenges, such as reading comprehension difficulties and the real-time cognitive processes underlying vocabulary learning and word recognition, both in native languages and foreign language acquisition [15,16]. However, despite the broad application of eye tracking technologies, the majority of studies utilizing these techniques to investigate language issues within ASD populations tend to concentrate on aspects related to native language processing and reading behaviors. Notably, there is a lack of research specifically employing eye tracking methods to examine language-related aspects in individuals with ADHD, indicating a significant gap in the current literature [11,17]. One of the most prominent approaches for studying language processing in eye tracking studies is the Visual World Paradigm (VWP), an experiment which was initially introduced by Cooper [18]. This method is utilized in psycholinguistic research and involves tracking participants’ eye movements as they look at images while listening to spoken language. Studies using the VWP have shown that certain eye movement patterns, such as the time taken to first fixation or the number of fixations on target stimuli, are strong indicators of successful word recognition [16,17,18,19,20,21].

Several studies have attempted to determine specific time limits that align with significant visual perception. In their research, Tanenhaus et al. [21] showed that individuals tend to focus their gaze on relevant objects within 200 to 1000 milliseconds after hearing a word. Other studies agree that early fixation on the correct target, typically occurring between 500 and 800 milliseconds after word onset, is linked to improved accuracy in word recognition and greater efficiency in language processing [19,20]. However, research has indicated that this time window is considerably longer in young children than that observed in adult populations, reflecting the ongoing developmental progression of lexical access mechanisms. In their study, Mani and Plunkett [22] explored how children aged between 18 and 24 months recognize words, utilizing the VWP. Their findings indicated that children typically focused accurately on the target image within 800 to 1200 milliseconds after the spoken word was initiated. Therefore, to accommodate variability in response times among young children, the researchers established a 1500-millisecond threshold as a pragmatic criterion for successful word recognition, applicable to familiar and newly acquired vocabulary. In addition, in the context of foreign language acquisition, studies have shown that even typically developing FL learners may exhibit delayed fixation patterns compared to native speakers [23].

Furthermore, distractor images sharing phonological similarities with the target words have also been shown to affect participants’ eye movements [24]. Specifically, when distractors possess names that phonologically resemble the target, participants frequently fixate on these distractors. Similarly, in a study by Hua et al. [25] that delved into semantically related distractors using the VWP with young children with ASD, it was found that autistic children, like their typically developing peers, were able to swiftly recognize words and understand their meanings. Nonetheless, autistic children tend to concentrate more on objects within the same category as the word (e.g., “cat” for the word “rabbit”) rather than on objects that are contextually related (e.g., “carrot” for “rabbit”). This pronounced emphasis on category-based (taxonomic) connections impeded their ability to concentrate on the correct object [25]. In addition, when it comes to non-native language speakers, a study by Soto & Schmid [26] revealed that individuals with higher vocabulary scores exhibit quicker and more accurate fixations on target words, whereas those with smaller vocabularies display slower responses and more frequent fixations on phonological competitors (e.g., confusing “carrot” with “parrot”). The researchers advocate for the previously described concept of a lexical threshold, suggesting that a certain level of vocabulary knowledge is essential for efficient spoken word recognition. Below this threshold, non-native language users have been shown to be more susceptible to distraction by similar-sounding or semantically plausible alternatives, indicating that limited lexical access renders word recognition more error-prone and less efficient [27].

Nevertheless, while it is evident that the VWP has been thoroughly utilized, it is crucial to acknowledge that the findings discussed mainly apply to typically developing individuals who process spoken words in their native language. Although there is an increasing amount of eye tracking research in both native and foreign language learning, neurodivergent learners are still largely overlooked in this area. To our knowledge, there is currently no available research exploring how the attentional differences associated with ADHD and ASD might influence visual processing and word recognition in the field of English as a Foreign Language (EFL) learning. Understanding the visual–cognitive mechanisms underlying word recognition in neurodivergent children is essential for designing more inclusive and effective foreign language interventions. Therefore, the present exploratory study seeks to address this gap by exploring visual word recognition thresholds in young EFL learners diagnosed with ADHD and ASD, using eye tracking technology within a VWP framework. Considering the exploratory nature of our study, we analyzed a wide array of gaze metrics that are prevalent in eye tracking research and are commonly applied in studies utilizing the VWP. Therefore, by examining several metrics, namely, time to first fixation (TTFF), time spent fixating (TSF), fixation count (FC), first fixation duration (FFD), and average fixation duration (AFD) on area of interest (AOI), the study aims to provide preliminary insights into the visual–attentional profiles of neurodivergent learners during foreign language vocabulary processing.

Thus, the study aims to explore the following questions:

Q1 Are there observable patterns in eye tracking metrics that relate to effective word recognition in young neurodivergent learners with ADHD and ASD, who are acquiring English as a foreign language?

Q2 Which types of distractors are most likely to interfere with accurate vocabulary recognition in young EFL learners with ADHD and ASD during a VWP task?

Q3 Do children with ADHD and children with ASD differ in their eye tracking measures during visual word recognition tasks in an EFL context?

Q4 Is there a relationship between eye tracking indicators of word recognition and learners’ (a) age and (b) prior vocabulary knowledge in the ADHD and ASD groups?

## 2. Materials and Methods

This study seeks to examine the eye gaze patterns of young learners with Attention Deficit/Hyperactivity Disorder (ADHD) and Autism Spectrum Disorder (ASD) during a real-time spoken word recognition task aimed at English as a Foreign Language (EFL) vocabulary recognition. To facilitate this investigation, a Visual World Paradigm was developed, incorporating 120 EFL vocabulary items at the beginner level. The materials, experimental design, and procedure are described in the following sections.

### 2.1. Experimental Apparatus

Initially, all lexical items included in the study were derived from the Cambridge Starters for Young Learners Vocabulary List (pre-A1). The selected words were age-appropriate, concrete, and highly imageable (e.g., “mouth”, “dog”, “car”), resulting in a total of 120 vocabulary terms. To evaluate prior vocabulary knowledge, a set of 120 flashcards was developed using a design application (Canva Pro) to illustrate the 120 terms included in the study. To evaluate the effectiveness of the flashcards in representing the depicted words, they were presented to a sample of ten typically developing native Greek children, aged 5 to 10 years. The children were instructed to identify, in Greek, the object or action depicted in the image. Any images deemed inappropriate were replaced until the responses stabilized. The images featured on the flashcards were also employed in the eye tracking experiment. The experiment assessed 44 lexical items/target images for their ability to be recognized in real-time. The 44 lexical items were chosen at random using a blind drawing technique from the 120 terms included in the study. The distractors were meticulously selected from the same set of words to ensure that each slide contained at least one phonologically and/or semantically related distractor, in addition to an unrelated image.

Furthermore, to avoid any potential bias linked to familiar voices, such as those of the researchers, a text-to-speech application (NaturalReader Online) was used to produce the audio that would be included in the VWP. The eye tracking equipment used in the study was the Tobii Pro X2-60 Hz system, which captures gaze positions at a sampling frequency of 60 Hz and achieves an approximate spatial accuracy of 0.5°. Data collection, stimulus presentation, and area of interest (AOI) management were facilitated through the iMotions 7.1 software platform. Finally, the experimental procedure was conducted on a Dell Vostro 15,500 series monitor, measuring 15.6 inches diagonally, with a resolution of 1024 × 768 pixels and a physical display dimension of 380 × 300 mm.

### 2.2. Participants

A total of seventeen children (n = 17) participated in this exploratory study, including nine children diagnosed with ADHD (n = 9), comprising seven boys and two girls, and eight children diagnosed with ASD (n = 8), comprising four boys and four girls. All the participants were native Greek speakers enrolled in an EFL program within a school setting. The participants’ ages ranged from 6 to 10 years (mean age = 8.35). The inclusion criteria for the study required participants to have a formal diagnosis of either ADHD or ASD and to possess a beginner (pre-A1) proficiency level in EFL. Following the acquisition of informed consent from all the participants’ parents or legal guardians, the students were administered the Greek version of the Raven’s Colored Progressive Matrices [28] to evaluate their non-verbal reasoning abilities and ensure a degree of cognitive homogeneity among the participants. This procedure aimed to control for significant variations in general cognitive functioning that could potentially confound the interpretation of the students’ word recognition performance. Concurrently, to confirm the diagnostic classifications and assess the severity of symptoms, the students’ parents completed two standardized screening instruments: the ADHD Rating Scale–IV [29] for individuals for ADHD and the Autism Spectrum Quotient [30] for ASD children. These instruments offered additional clinical insights beyond the formal diagnosis, facilitating the precise categorization of the participants based on attentional and social–communicative profiles. Moreover, the participants’ proficiency in English was measured using the Cambridge Young Learner’s Placement Test [31]. Lastly, parents were asked to complete a demographics questionnaire to obtain information about the child’s age, biological sex, family socioeconomic status, and any previous experiences with language learning.

In the ADHD Rating Scale-IV [29], the group of 9 children diagnosed with ADHD had an average total score of 38.22 (SD = 6.45), with individual scores ranging from 29 to 48. The mean score for the Inattention subscale was 20.11 (SD = 3.40), while the Hyperactivity/Impulsivity subscale had a mean of 18.11 (SD = 4.01). All the participants surpassed the clinical threshold, thereby confirming a significant level of ADHD-related symptomatology. The internal consistency was found to be excellent, with a Cronbach’s α of 0.91. The mean total score on the AQ-Child within the current sample (n = 8) was 109.25 (SD = 10.46), with individual scores spanning a range from 94 to 123. Notably, all the participants exceeded the established cut-off for clinical significance. Based on the Greek adaptation of the AQ-Child [30], a score of 76 or higher is indicative of clinically significant traits, as determined by Greek normative data. The internal consistency of the AQ-Child in this sample was strong, demonstrated by a Cronbach’s alpha coefficient of 0.85.

The mean raw score of all the children on the Raven’s Colored Progressive Matrices [28] was 28.94 (SD = 3.12), with scores ranging from 24 to 35. According to age-normed Greek standards, 88% of the children (15 out of 17) scored within the average or above-average range (standard scores between 90–115), while 2 children (12%) scored in the below-average range (80–89). No participant fell within the intellectual disability range (i.e., standard score < 70). These results confirm adequate non-verbal cognitive functioning across the sample. Ultimately, all the participants were classified as having beginner-level proficiency in English according to the English language proficiency test. The demographic data further confirmed that all the participants originated from urban or semi-urban areas, with the parental reports indicating comparable levels of education and access to English instruction within school settings. The parents did not report a prior diagnosis of intellectual disability or speech–language disorder, such as dyslexia, or any other co-occurring condition for any of the children participating, thereby ensuring the sample met the study’s inclusion criteria.

### 2.3. Procedure

Once eligibility for participation was confirmed, the participants underwent a pretest assessment to evaluate their prior knowledge of the English vocabulary included in the VWP. During the pretest phase, the participants were presented with 120 flashcards, organized into ten rounds of 12 words each. These flashcards depicted the vocabulary utilized in the experiment, encompassing both the target images and distractors. The researcher called out a word and the participants were asked to point to the corresponding image from the flashcards presented in front of them. All correct responses were marked on a checklist.

The eye tracking experiment was conducted on a different day than the pretest assessment. During that time participants were invited to the Bilingual Education Lab of the University of Thessaly and were individually assessed. The participants were seated approximately 50 cm from the screen in a well-lit, quiet environment. Before the experiment commenced, the participants were informed of its objectives and were explicitly instructed regarding their tasks and the expectations placed upon them. Additionally, they were shown a brief segment of a mock trial to familiarize themselves with the experimental setup. The experiment was divided into three sessions, each lasting approximately one and a half minutes, while short breaks were provided between sessions to reduce fatigue. A 9-point calibration procedure was performed before each session, and recalibration occurred whenever the validation error exceeded 1° of visual angle.

**Figure 1:**
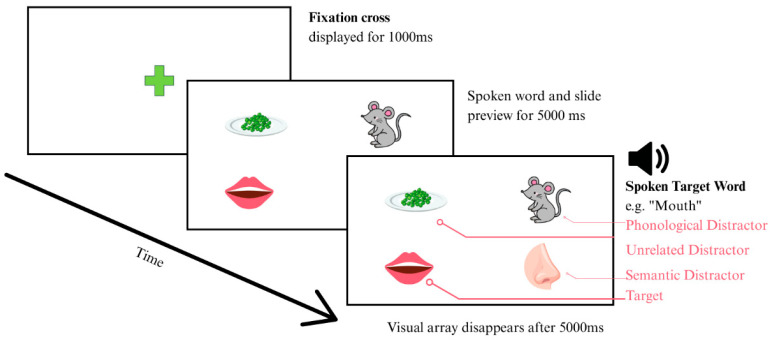
Sample trial of the Visual Word Paradigm.

The first session included 14 trials, while the subsequent sessions each contained 15 trials. Since each trial assessed a single target word, a total of 44 vocabulary items were tested per participant, selected from the original pool of 120 items. Each trial started with the presentation of a fixation cross at the center of the screen for a duration of 1000 milliseconds. This was followed by a word recognition slide displaying four images: one target image and three distractor images, concurrent with the auditory presentation of the target image. The phonological distractors shared onset or rhyme with the target (e.g., “mouth” and “mouse”) or began with the same phoneme (e.g., “bed” and “bath”), the semantic distractors were from the same category (e.g., “mum” and “grandma”), and the unrelated distractors had no perceptual or linguistic overlap, ensuring contrast. The images were displayed for 5000 milliseconds (see Figure 1). The participants were instructed to focus on the image corresponding to the auditory cue and to maintain their gaze until the subsequent fixation cross appeared.

### 2.4. Data Preprocessing

This study focused on several eye tracking metrics within specified areas of interest (AOIs) and more specifically on the following metrics: (a) time to first fixation (TTFF), i.e., the time it takes for a participant to first fixate on the target stimulus after it appears; (b) time spent fixating (TSF), i.e., the total duration of all fixations on the target; (c) fixation count (FC), i.e., how many times the participant fixated on the target area; (d) first fixation duration (FFD), i.e., the length of the initial fixation on the target, and (e) average fixation duration (AFD), i.e., the mean duration of all fixations on the target. Each participant was exposed to 44 stimuli. However, due to significant hyperactivity, one participant with ADHD could not complete the last two sessions, so only the eye tracking data from the initial session was used for this individual (n = 14 trials). Before proceeding with the data analysis, we performed several preprocessing steps. Initially, we discarded trials that had incomplete or invalid eye tracking data. Specifically, any trial where all the primary eye movement metrics were recorded as zero was eliminated from the dataset. Such instances likely reflected participant disengagement, or technical malfunctions that prevented the capture of fixation data within the target AOI. This procedure ensured that only trials exhibiting considerable gaze behavior were retained for the purposes of threshold determination and subsequent analyses.

Moreover, in order to establish empirically validated thresholds indicative of successful word recognition, percentile analyses were conducted, incorporating only those stimuli that involved the target AOI. Additionally, only stimuli that the participants had correctly identified in the pretest were included, as the absence of prior recognition in the pretest precluded any assumption of recognition during the eye tracking experiment. Following the calculation of percentiles, the dataset was subsequently aggregated, such that each participant was represented by a single central tendency measure (median) for each AOI category and the phonological, semantic, and unrelated distractors. This process yielded a repeated-measures data structure, with each row corresponding to a participant and separate variables denoting each AOI and associated metric.

### 2.5. Statistical Analysis

Considering the non-normal distribution observed in the fixation measures, non-parametric statistical methods, including the Friedman test, Wilcoxon signed-rank test, Mann–Whitney U test, and Spearman’s rank correlation, were utilized for group comparisons. This analytical strategy aligns with contemporary methodological guidelines for the analysis of Visual World Paradigm data, particularly when focusing on aggregated fixation metrics rather than continuous time-course measures [32]. Percentiles from 719 trials at 10%, 25%, 75%, and 90% were computed to establish the lower and upper limits of gaze behavior linked to precise word recognition. The calculation of percentiles is a common practice in eye tracking research [33,34]. This methodology facilitates the classification of gaze behavior without presupposing normality in the data distribution. A series of Friedman tests, serving as the non-parametric counterpart to repeated-measures ANOVA, was employed to compare metrics such as TTFF, FC, and TSF across the three distractor types (semantic, phonological, unrelated). This analysis facilitated the evaluation of whether specific distractor types systematically elicited earlier or prolonged visual attention. In the event of a significant Friedman test result, subsequent pairwise comparisons were planned using Wilcoxon signed-rank tests to identify differences between distractor pairs.

Furthermore, to examine potential differences in visual attention patterns between participants with ADHD and ASD mean values of TTFF, FFD, TSF, FC, and AFD, were computed across all trials where the target word was recognized correctly. Given the limited sample size and the deviations from normality found in the descriptive statistics of several variables, the Mann–Whitney U tests were employed for comparing groups. Finally, in order to explore how developmental factors and prior vocabulary knowledge might affect visual attention during word recognition, non-parametric correlation analyses were conducted to evaluate the link between the participants’ age and their combined eye tracking metrics. Specifically, Spearman’s rank-order correlation coefficients were calculated to investigate the connections between age and each of the following variables: TTFF, FFD, TSF, FC, and AFD. This approach aimed to determine whether the older children demonstrated more rapid or efficient gaze patterns during English as a Foreign Language (EFL) vocabulary processing. All analyses were conducted using the Statistical Package of Social Sciences (SPSS 29.0.2).

## 3. Results

The following section presents analyses of the eye tracking data with the aim of exploring visual attention patterns during spoken word recognition in children diagnosed with Attention Deficit/Hyperactivity Disorder (ADHD) and Autism Spectrum Disorder (ASD). The analyses included percentile-based thresholds for successful word recognition, distractor-type effects, and between-group comparisons and correlations.

Table 1 presents the percentile-based criteria for successful word recognition, categorized into groups of students diagnosed with ADHD and ASD.

Initially, two percentile-based thresholding strategies were implemented to address the inter-individual variability in neurodivergent populations and separate threshold values were calculated for each group to respect the cognitive and attentional differences between the ADHD and ASD participants:

1. High-Confidence Recognition Zone (25th–75th percentile):
  This range encompasses the interquartile distribution of performance and can be utilized to identify trials that exhibit the most stringent fixation behavior patterns associated with recognized word identification.
2. Feasible Recognition Window (10th–90th percentile):
  This range allows for greater inclusivity and reflects realistic variation in cognitive processing, especially within clinical populations. Trials falling within this window are considered to exhibit plausible patterns of recognition, even if they deviate slightly from the median behavior.

**Figure 2:**
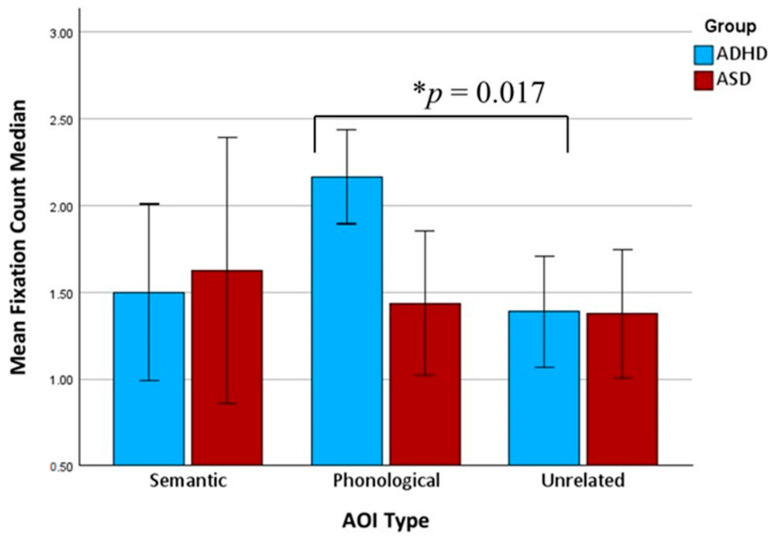
Average of participant-level median fixation count (±95% CI) across semantic, phonological, and unrelated distractors in ADHD and ASD groups. Asterisk indicates a significant difference in fixation count between phonological and unrelated distractors in the ADHD group (*p* = 0.017, Wilcoxon signed-rank test, Bonferroni-adjusted).

**Figure 3:**
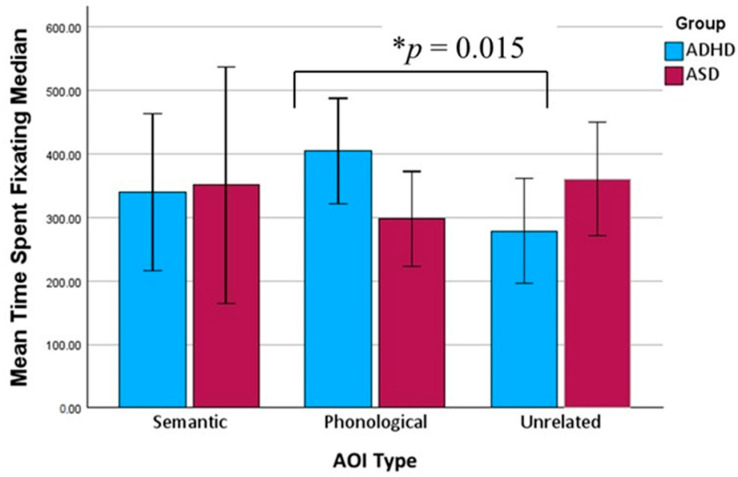
Average of participant-level median time spent (±95% CI) across semantic, phonological, and unrelated distractors in ADHD and ASD groups. Asterisk indicates a statistically significant difference in fixation duration between phonological and unrelated distractors in the ADHD group (*p* = 0.015, Wilcoxon signed-rank test, Bonferroni-adjusted).

Friedman tests were conducted to examine differences in gaze behavior across the three distractor types (semantic, phonetic, and irrelevant) within each group. No significant differences in time to first fixation (TTFF) were found for either the ADHD group or the ASD group, indicating that the distractor type did not significantly affect initial gaze orientation. However, the ADHD group showed significant effects for both time spent fixating [χ^2^(2) = 8.22, *p* = 0.016] and fixation count [χ^2^(2) = 9.74, *p* = 0.008; see Figure 2 and Figure 3], with increased gaze engagement for the phonologically related distractors compared to the unrelated ones. The detailed pairwise results are reported below.

Follow-up Wilcoxon signed-rank tests (Bonferroni-adjusted α = 0.017) revealed that the participants with ADHD spent significantly more time on the phonologically related distractors than the unrelated distractors (Z = −2.43, *p* = 0.015) and had significantly more fixations on the phonologically related distractors than on the unrelated distractors (Z = −2.39, *p* = 0.017). The other pairwise comparisons were not statistically significant. In contrast, the ASD group did not show significant differences across the distractor types for either time spent or fixation count.

Subsequently, the Mann–Whitney U tests, which were employed to formally compare the ADHD and ASD groups on all the fixation metrics, showed no significant differences and all the effect sizes were small across all the metrics. To examine the relationships among the eye tracking metrics, age, and vocabulary proficiency, Spearman’s rank-order correlation analyses were conducted independently for the ADHD and ASD groups. In the ADHD group (n = 9), age was significantly positively correlated with fixation count (r_s_ = 0.70, *p* = 0.035) and with the vocabulary pretest scores (r_s_ = 0.80, *p* = 0.009). Moreover, a strong positive correlation was observed between pretest scores and fixation count (r_s_ = 0.88, *p* = 0.002). In the ASD group (n = 8), age was significantly associated with both time spent fixating (r_s_ = 0.78, *p* = 0.023) and the pretest scores (r_s_ = 0.72, *p* = 0.042). Time spent fixating was also positively correlated with fixation count (r_s_ = 0.89, *p* = 0.003) and average fixation duration (r_s_ = 0.81, *p* = 0.015). Additionally, a strong correlation was found between average and first fixation duration (r_s_ = 0.91, *p* = 0.002).

## 4. Discussion

This exploratory study addressed four primary research questions concerning the visual word recognition processes of young EFL learners with ADHD and ASD. First, clear patterns in eye tracking metrics were identified (Q1), with successful word recognition associated with gaze behavior falling within defined percentile thresholds for metrics such as fixation count and time spent fixating. These findings suggest that percentile-based gaze thresholds can serve as a practical benchmark for evaluating recognition efficiency in neurodivergent populations. Second, the phonologically related distractors were found to interfere most with accurate vocabulary recognition in the ADHD group (Q2), as evidenced by significantly more fixations and longer fixation durations compared to the unrelated distractors. The children with ASD, by contrast, showed no significant differences across the distractor types. Third, no significant group differences (Q3) were found between the ADHD and ASD participants in their overall eye tracking patterns, suggesting similar engagement strategies during the EFL recognition task. Finally, both age and prior vocabulary knowledge (Q4) were positively associated with more efficient and deliberate gaze behavior in both groups, supporting developmental links between lexical knowledge and visual attention strategies during language learning.

Regarding the first research question, this study delineates both narrow (25th–75th percentile) and broad (10th–90th percentile) recognition zones, which may serve as a potential benchmark for future research and intervention strategies. We propose using the 10th–90th percentile threshold to accommodate the individual variations among children within these populations and recommend employing two or more metrics, such as fixation count and time spent on the target area of interest, to effectively determine word recognition within the VWP based on these thresholds. Finally, apart from establishing thresholds, the calculated percentiles indicated that both groups exhibited comparable gaze patterns during the accurate recognition of words, as evidenced by the largely overlapping percentile ranges for TTFF, TSF, FC, FFD, and AFD. Furthermore, the gaze patterns observed in children with ADHD and ASD differ from those identified in previous studies of typically developing children. Research involving younger children indicates that the time to first fixation on a target image occurs between 1000–1200 ms [22]. However, our findings reveal that word recognition occurs at approximately 1300 ms at the 75th percentile for both groups of children. Given that this study involves a foreign language, it implies the necessity for more lenient thresholds compared to those established for typically developing children. To our knowledge, this research constitutes one of the first efforts to employ percentile-based eye tracking thresholds for assessing word recognition performance among children with ADHD and ASD in the context of learning English as a Foreign Language (EFL) vocabulary.

In addressing our second inquiry, “Which types of distractors are most likely to interfere with accurate vocabulary recognition in young EFL learners with ADHD and ASD during a VWP task?”, subtle distinctions were identified concerning the impact of the distractor type. Notably, the children with ADHD demonstrated markedly higher fixation counts and extended fixation durations on the phonologically related distractors compared to both the semantic and unrelated distractors. This pattern aligns with existing research on the processing of stimuli that share phonological characteristics with the target word and suggests that children with ADHD exhibit similar patterns to typically developing (TD) individuals [24]. In contrast, the children with ASD exhibited no significant differences across distractor types in any gaze metric, suggesting a more uniform or rigid scanning strategy that is less influenced by phonological or semantic similarity. Although the children with ASD did not exhibit increased fixation on semantically related distractors in the current study, this finding should be interpreted with caution.

Moreover, regarding our third research question, “Do children with ADHD and children with ASD differ in their eye tracking measures during visual word recognition tasks in an EFL context?”, there were no significant differences between the ADHD and ASD groups across any of the fixation metrics examined. Moreover, all the observed effect sizes were small, suggesting minimal practical differences in visual attention patterns between the two groups during the EFL word recognition task. This outcome implies that, despite their distinct neurocognitive profiles, children with ADHD and ASD may exhibit broadly comparable overall gaze behaviors when recognizing spoken vocabulary in a structured experimental setting.

Finally, regarding our fourth research question, “Is there a relationship between eye tracking indicators of word recognition and learners’ age and prior vocabulary knowledge in young neurodivergent English language learners?”, the correlation analyses revealed meaningful developmental patterns in gaze behavior and vocabulary knowledge among both the ADHD and ASD groups, consistent with findings from previous research. In the ADHD group, older children tended to have higher vocabulary scores and made more fixations during the task, suggesting that as age and lexical knowledge increase, so does the efficiency of visual search and engagement with target stimuli. This aligns with studies showing that vocabulary growth enhances word recognition speed and accuracy over time [23]. The strong association between vocabulary scores and fixation count also supports the idea that children with better lexical knowledge in the non-native language engage more actively with the visual scene, likely using fixations as a strategy to confirm word-image matches [26].

In the ASD group, age was positively associated with both vocabulary knowledge and the time spent fixating on relevant stimuli, indicating a developmental trend toward more sustained visual engagement as children grow older. This is consistent with research suggesting that although children with ASD may exhibit atypical attention patterns early on, they can show improvements in sustained attention and language processing with age and increased exposure [35]. The strong correlations between time spent fixating, fixation count, and fixation durations suggest that children with ASD who spend more time attending to the visual input also exhibit more stable and deliberate gaze behavior, possibly reflecting deeper processing of the auditory-visual associations. Collectively, these findings support the notion that both age and vocabulary knowledge play a crucial role in shaping visual attention during word recognition, even in neurodivergent populations [27].

### Limitations

In summary, although this study provides initial insights into the word recognition processes of young ADHD and ASD EFL learners, it is important to acknowledge several limitations. Firstly, the relatively small sample size, particularly within each diagnostic group, limits the statistical power and generalizability of the findings. Although the exploratory nature of the analysis is appropriate for an initial investigation, replication in larger and more diverse samples is necessary to ensure robustness. Secondly, the lack of a typically developing control group prevents direct comparisons with normative developmental patterns in visual attention during foreign language processing.

Furthermore, while eye tracking provides valuable insights into learners’ visual attention and processing patterns during word recognition tasks, it does not, on its own, guarantee that a participant has successfully understood or recognized a word. As researchers have pointed out, relying exclusively on eye movement data can lead to overinterpretation of cognitive processes, especially in language learning contexts. Scholars highlight the importance of triangulating eye tracking data with complementary behavioral measures, such as mouse clicks, verbal translations, or recall tasks, to more reliably confirm that a fixation corresponds to actual word comprehension [36,37]. Future research would therefore benefit from incorporating a secondary confirmation step apart from the pretest to ensure that the observed fixation behavior truly reflects successful word learning and not mere visual orientation.

Finally, previous research has shown that autistic individuals tend to focus more on categorically or taxonomically related items, such as grouping animals together, rather than on thematically or contextually related items [25]. In our study, the term “semantic distractors” encompassed both taxonomic and thematic relationships, which may have masked the specific patterns of attention that are more characteristic of autistic learners. It is therefore possible that the absence of a significant difference in gaze behavior toward semantic distractors reflects the heterogeneous nature of the semantic relationships included, rather than a lack of sensitivity to semantic content altogether. Future studies should consider disentangling taxonomic from thematic distractors to more precisely capture the semantic processing profiles of children with ASD during language comprehension tasks.

## 5. Conclusions

The findings of this study have substantial implications for the formulation of foreign language teaching strategies specifically designed for neurodivergent learners. The establishment of percentile-based eye tracking thresholds offers an initial framework for evaluating variability in word recognition efficiency, which could guide the development of personalized instructional pacing or the incorporation of adaptive learning technologies. Furthermore, the heightened susceptibility of children with ADHD to phonologically similar distractors highlights the importance of minimizing phonological interference in vocabulary instruction. This may necessitate the careful selection of word lists or a reduction in the simultaneous presentation of phonologically similar items. By acquiring a comprehensive understanding of the distinct attentional and cognitive processing profiles that characterize neurodivergent students, educators, and intervention designers can tailor foreign language curricula to accommodate individual differences, thereby improving the efficacy of vocabulary acquisition.

## Figures and Tables

**Table 1:** Eye tracking thresholds for successful word recognition by group (TTFF, TSF, FFD, AFD in ms).

Group	Percentile	TTFF	TSF	FC	FFD	AFD
ADHD	**10**	150.50	380.00	2.00	107.00	156.50
	**25**	431.75	832.50	4.00	124.00	190.25
	**75**	1344.25	2695.50	8.00	279.25	332.50
	**90**	2100.50	3417.00	11.00	419.00	465.50
ASD	**10**	162.00	264.00	1.00	111.00	140.00
	**25**	540.50	641.50	3.00	136.00	180.50
	**75**	1347.00	2837.00	9.00	270.00	322.50
	**90**	1986.00	3685.00	11.00	436.00	468.00

**Note.** TTFF = time to first fixation; TSF = time spent fixating; FFD = first fixation duration; AFD = average fixation duration. All time-based measures are in milliseconds (ms). FC = fixation count (unitless). Percentiles are based on data from word recognition instances where participants correctly identified the word in the pretest.

## Data Availability

The datasets generated and analyzed during the current study is available from the corresponding author upon reasonable request due to privacy.
